# Freestanding Bamboo‐Like Nitrogen‐Doped Carbon Nanofibers/PANI Dual‐Conductive Cathodes via Interfacial Engineering for High‐Performance Lithium–Sulfur Batteries

**DOI:** 10.1002/advs.76380

**Published:** 2026-06-27

**Authors:** Jie Yang, Fan Wang, Enci Wang, Haile Qian, Terence Xiaoteng Liu, Jiarui Huang

**Affiliations:** ^1^ College of Chemistry and Materials Science Key Laboratory of Functional Molecular Solids of the Ministry of Education Anhui Normal University Wuhu Anhui P. R. China; ^2^ School of Materials Science and Engineering Tongling University Tongling Anhui P. R. China; ^3^ School of Engineering Physics and Mathematics Faculty of Science and Environment Northumbria University Newcastle upon Tyne UK

**Keywords:** bamboo‐like, cathode, Li−S batteries, nitrogen‐doped carbon nanofibers, polyaniline

## Abstract

Lithium–sulfur (Li–S) batteries are fascinating next‐generation energy storage devices because of their high energy density, but they face problems such as polysulfide (LiPS) shuttling and sluggish reaction kinetics. Herein, a freestanding film consisting of ultralong bamboo‐like nitrogen‐doped carbon nanofibers (BNCFs) is developed using melamine as a raw material and aluminum foil as a catalyst to form Al_4_C_3_ nanoparticles via a vapor‐liquid‐solid growth process at high temperature. Subsequently, the BNCFs are combined with sulfur and in situ coated with polyaniline (PANI) to form a freestanding binder‐free cathode (BNCFs/S/PANI). The freestanding and binder‐free BNCFs film creates orderly channels for polysulfide adsorption, volume buffering, and efficient Li^+^/electron transport. The PANI coating chemically connects LiPSs, enhances uniform lithium deposition, and elevates redox activity via its quinonediimine and phenylenediamine units. This synergistic effect endows the cathode with superior cycling stability, leading to a minimal capacity decay rate of merely 0.047% per cycle over 500 cycles at 1.0 A g^−1^. This research presents a new cathode design for high‐energy‐density Li–S batteries.

## Graphical abstract

Bamboo‐like nitrogen‐doped carbon fibers (BNCFs) were synthesized via a vapor‐liquid‐solid catalytic growth method and further integrated with polyaniline to fabricate a freestanding BNCFs/S/PANI cathode. The PANI coating and BNCFs framework synergistically establish an interconnected conductive network with abundant nitrogen‐active sites, thereby significantly enhancing sulfur redox kinetics. Moreover, the polysulfide shuttle effect is effectively suppressed through a dual physical‐chemical confinement mechanism.

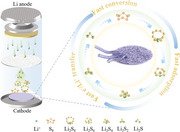

## 1 Introduction

As the global population continues to increase, the demand for green, sustainable, and renewable energy sources is growing rapidly [1, 2]. Lithium–sulfur (Li–S) batteries, as next‐generation energy storage systems, have drawn significant attention due to their ultrahigh theoretical energy density and the cost‐effectiveness of sulfur resources [3, 4, 5, 6]. However, the insulating nature of sulfur cathodes and their discharge products (Li_2_S_2_/Li_2_S), severe polysulfide shuttling, and enormous volume expansion (∼80%) during cycling result in poor sulfur consumption, immediate capacity fading, inferior cycling stability, and low Coulombic efficiency [7, 8, 9]. These inherent drawbacks have severely hindered the widespread commercial application of Li–S batteries.

To overcome the above issues, a variety of solutions have been suggested, including altering sulfur cathode composition/architecture, inventing functional binders, engineering superior separators, and using electrolyte additives [10, 11, 12, 13, 14]. In the past few decades, substantial research has focused on designing advanced sulfur host materials to enhance Li–S battery efficiency [15]. Carbon‐derived materials with a large specific surface area and excellent conductivity, such as porous carbon sheets, carbon nanofibers, carbon nanotubes, and graphene, have been extensively investigated as sulfur hosts [16, 17, 18, 19].

Particularly, micro/nanostructured carbon materials not only elevate electronic conductivity and sulfur consumption but also mitigate the shuttle effect to some extent while providing more active sites to facilitate the adsorption of lithium polysulfides (LiPSs) [20]. However, low‐dimensional nanostructures tend to agglomerate owing to van der Waals forces, hindering electrolyte penetration. Moreover, nonpolar carbon materials rely solely on weak van der Waals interactions to regulate polar LiPS, which are inadequate to hinder LiPSs’ migration, resulting in delayed redox kinetics. The one‐dimensional (1D) structures and sp^2^ carbon hybridization of carbon nanotubes and carbon fibers endow them with outstanding electrical conductivity, mechanical durability, and chemical stability [21, 22].

To reduce the severe shuttle effect and boost redox kinetics, heteroatoms (N, O, P, S) have been incorporated into carbon‐based materials to enhance their polarity and sulfur affinity, thereby boosting the adsorption of LiPSs [23, 24]. In particular, N, O, and S heteroatoms possess fairly high electronegativity, enabling significant interactions with lithium in long‐chain LiPS species (Li_2_S_4‐8_) [25]. Tian et al. reported a 3‐D N‐doped Murray carbon nanostructure coated with a polydopamine (PDA) layer (P@S/N‐MCN) as a sulfur host, where N heteroatoms enhance chemical adsorption to anchor active sulfur species [26]. However, achieving adequate catalytic efficiency typically requires a high catalyst loading, which may drastically reduce the overall energy density of Li–S batteries.

Conventional carbon/sulfur cathodes utilize heavy metal current collectors (such as aluminum foil), conductive agents, and binders. As the sulfur loading increases, the electrode thickens, resulting in long transport channels and high resistance to lithium‐ion (Li^+^) diffusion [27]. To address these drawbacks, flexible freestanding carbon/sulfur cathodes have gained significant popularity because of their binder‐free nature, high conductivity, and bendability [28, 29]. Additionally, the interconnected current collectors enable continuous high‐speed transport pathways for electrons and ions, thereby enhancing sulfur usage and redox reaction kinetics [30]. Guo et al. described a flexible composite sulfur cathode consisting of carbon fibers, sulfur‐immobilized carbon nanotubes, and porous reduced graphene oxide, which integrates both diffusion‐limiting and dissolution‐blocking abilities [31]. Meanwhile, Wang et al. synthesized an N,O co‐doped 3‐D wood‐like carbon framework adorned with vertically arranged carbon nanotube forests via an ice‐templating method [32]. This structure exhibits high conductivity and wood‐inspired structural stability, permitting high sulfur loading as an efficient host. However, inorganic nitrogen‐doped carbon materials may catalyze side reactions between polysulfides and the electrolyte, accelerating self‐discharge and capacity deterioration [33]. By contrast, organic compounds such as polyaniline (PANI) [34], polypyrrole (PPy) [35], and polythiophene (PTH) [36], with their numerous chemical bonds and functional groups, can greatly improve the cycling performance of Li–S batteries.

Specifically, PANI's amine‐rich surface enables strong Lewis acid‐base interactions with LiPSs, severely limiting their shuttling between the cathode and the anode [37]. Furthermore, its conjugated structure can be further expanded through the incorporation of heteroatoms (protic acids, inorganic metal compounds, and other inherently conductive molecules), thereby boosting overall conductivity [38]. Li et al. deposited polyaniline onto a scaffold composed of reduced graphene oxide and carbon nanotubes (denoted as iPANI@rGO‐CNT) [39]. This composite architecture combines high conductivity, lithiophilicity, and a porous nanostructure, facilitating uniform Li^+^ deposition and successfully inhibiting lithium dendrite growth. Consequently, incorporating nitrogen‐rich conductive organic PANI into nitrogen‐doped carbon materials can simultaneously address the issues of polysulfide shuttling, dendrite growth, and sluggish redox kinetics.

Herein, aluminum foil is used as a catalyst to form Al_4_C_3_ nanoparticles as dynamic catalytic centers, nitrogen–carbon precursors generated from melamine pyrolysis are driven to form bamboo‐like nitrogen‐doped carbon fibers (BNCFs), which continuously grow and spontaneously interweave into a network film. This approach enables the fabrication of a free‐standing cathode that does not require additional binders or current collectors. After sulfur loading, a thin conductive polyaniline (PANI) layer is in situ coated onto the surface of BNCFs, leading to a flexible 3‐D self‐supporting cathode material (BNCFs/S/PANI) for Li–S batteries. Without the addition of polymer binders or current collectors, the nitrogen‐doped carbon nanofibers provide adequate space and suitable surfaces for the rapid transfer of Li^+^/electrons, physical confinement of LiPSs, and accommodation of electrode volume expansion, maintaining high sulfur loading and minimizing volume fluctuations. The PANI, with its lone pair of electrons, not only forms chemical bonds with the sulfur in LiPSs to adsorb polysulfides on its surface but also exhibits lithiophilic properties, facilitating uniform lithium deposition and avoiding lithium dendrite development. Furthermore, the PANI structure, rich in both phenylenediamine and quinonediimine units, possesses distinct redox properties that participate in electrochemical reactions, providing additional faradaic capacitance and boosting battery stability. Additionally, the combination of BNCFs with in situ coated PANI creates a dual‐confinement strategy (physical confinement by the hollow carbon framework and chemical adsorption by PANI) that has not been reported for bamboo‐like carbon nanofibers. The reversible redox activity of PANI's quinonediimine/phenylenediamine units provides additional catalytic functionality beyond simple N‐doping. Thus, the developed self‐supporting BNCFs/S/PANI cathode material provides valuable insights into the production of a high‐performance Li–S battery.

## 2 Results and Discussion

### 2.1 Material Design and Preparation

**FIGURE 1:**
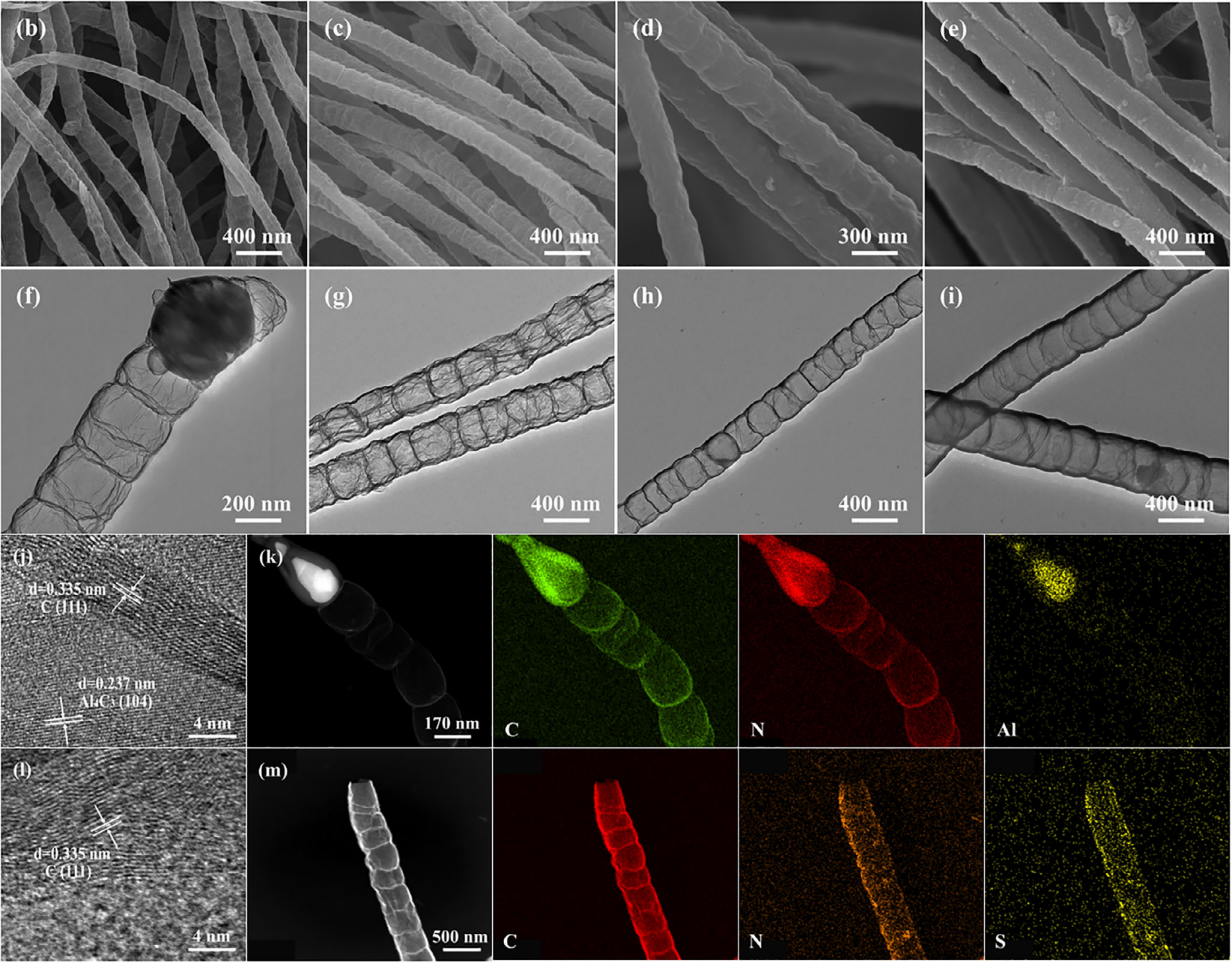
(a) Schematic of the synthesis process of BNCFs/S/PANI. SEM images of (b) BNCFs‐Al_4_C_3_, (c) BNCFs, (d) BNCFs/S, and (e) BNCFs/S/PANI. TEM images of (f) BNCFs‐Al_4_C_3_, (g) BNCFs, (h) BNCFs/S, and (i) BNCFs/S/PANI. (j) HRTEM image of BNCFs‐Al_4_C_3_. (k) TEM image and corresponding elemental mapping images of BNCFs‐Al_4_C_3_. (l) HRTEM image of BNCFs/S/PANI. (m) TEM image and corresponding elemental mapping images of BNCFs/S/PANI.

Figure 1a illustrates the synthetic steps for BNCFs with a uniform structure. Initially, using metallic aluminum as the catalyst and melamine as both carbon and nitrogen source, the BNCFs are grown in an inert atmosphere at 1200°C via a vapor‐liquid‐solid (VLS) growth process. The Al_4_C_3_ phase formed through the interaction between aluminum and carbon‐nitrogen species is encapsulated by carbon layers. After eliminating Al_4_C_3_ via aqua regia, the resulting BNCFs have a greatly enhanced specific surface area and numerous active functional groups including –OH and –COOH. BNCFs/S composites were manufactured via a sulfur melt‐diffusion process, followed by in situ PANI coating to yield free‐standing BNCFs/S/PANI films (12 mm in diameter). BNCFs have high mechanical flexibility, with folding tests demonstrating well‐maintained structural integrity, allowing them to be used directly as current‐collector‐free cathodes for Li–S batteries (Figure S1).

The microstructure of the products at various phases was visually examined using scanning electron microscopy (SEM) and transmission electron microscopy (TEM). The SEM images show that the ultralong bamboo‐like BNCFs‐Al_4_C_3_ have a multi‐walled carbon nanotube structure and form a 3‐D interconnected network, indicating improved electron transport pathways (Figure 1b and Figure S2a). TEM examination verifies its hollow structure, with Al_4_C_3_ located at the tips of BNCFs cavities, and a carbon shell thickness of about 10 nm (Figure 1f and Figure S2e). HRTEM, elemental mapping, and EDS investigations reveal the existence of both C and Al_4_C_3_ in the material (Figure 1j,k, and Figure S4a). The lattice fringe spacings of 0.237 and 0.335 nm correspond to the (104) crystal plane of Al_4_C_3_ and the (111) plane of graphitic carbon, respectively, demonstrating the effective conversion of melamine into BNCFs‐Al_4_C_3_ catalyzed by Al. Elemental mapping further indicated a homogeneous distribution of C and N elements throughout the BNCFs, suggesting excellent compositional uniformity of the composite.

Meanwhile, the Al element exists only in the form of nanoparticles within the carbon nanotubes. After aqua regia treatment, the BNCFs develop enhanced surface roughness and form a wrinkled hierarchical structure (Figures 1c and S2b). The interwoven hierarchical carbon film produces a strong, highly conductive carbon framework while enhancing the quantity of oxygen‐containing functional groups, thereby preventing polysulfide shuttling [18]. TEM images indicate that the hollow bamboo‐like structure is well retained after acid treatment, and the expanded length facilitates long‐range charge transfer during charge/discharge processes (Figures 1g and S2f). HRTEM and EDS analysis verified the complete elimination of Al_4_C_3_ (Figures S2i,j and S4b). After sulfur compositing, the BNCFs/S surface appears smooth (Figures 1d and S2c), and TEM reveals homogeneous sulfur filling within the carbon fiber cavities, demonstrating uniform sulfur‐BNCFs integration (Figures 1h and S2g). No‐agglomeration structures are observed in the in situ polymerized PANI (Figures 1e and S2d). The formation of a uniform coating is observed at an optimized aniline dosage (15 µL), while an insufficient dosage leads to incomplete coverage, and an excessive dosage results in an overly thick PANI layer that adversely affects mass transport and electrolyte infiltration (Figure 1i and Figures S2h and S3). HRTEM (Figure 1l) identified 0.335 nm lattice fringes corresponding to the (111) crystal plane of graphitic carbon in BNCFs/S/PANI. Elemental mapping (Figure 1m and Figure S4c) confirmed the homogeneous distribution of C, N, O, and S within the hollow fibers.

**FIGURE 2:**
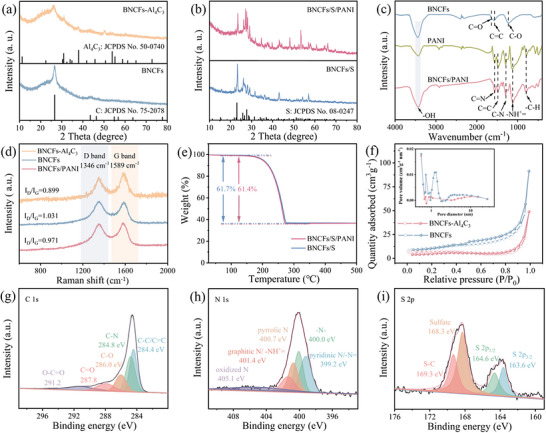
(a) XRD patterns of BNCFs‐Al_4_C_3_ and BNCFs, and (b) BNCFs/S and BNCFs/S/PANI. (c) FT‐IR spectrum of PANI, BNCFs, and BNCFs/PANI. (d) Raman spectrum of BNCFs‐Al_4_C_3_, BNCFs and BNCFs/PANI. (e) TGA curves of BNCFs/S and BNCFs/S/PANI. (f) N_2_ adsorption/desorption isotherm of BNCFs‐Al_4_C_3_ and BNCFs. The insets are the corresponding pore size distributions. High‐resolution XPS spectra of BNCFs/S/PANI: (g) C 1s, (h) N 1s, and (i) S 2p.

SEM and TEM results reveal that the BNCFs‐Al_4_C_3_ exhibit a uniform bamboo‐like hollow structure with an internode spacing of approximately 300 ± 20 nm. Moreover, Al_4_C_3_ is encapsulated within the carbon layers, indicating that the BNCFs grow via a VLS mechanism driven synergistically by melamine pyrolysis and molten aluminum nanoparticles. EDS mapping shows localized enrichment of Al at the nodes, while C and N are continuously distributed along the fiber axis, suggesting that Al_4_C_3_ acts as dynamic catalytic centers, regulating the deposition of carbon and nitrogen species as well as structural formation. Specifically, at 800°C–950°C, C_3_N_4_ generated from the pyrolysis of melamine in the lower temperature range is selectively adsorbed and catalytically cracked by Al_4_C_3_ particles, leading to the formation of graphene‐like layered structures at the liquid–solid interface (Figure 1f). As carbon layers continue to accumulate, the Al_4_C_3_ particles migrate forward due to the combined forces of surface tension and gravity, exposing novel catalytically active surfaces for additional carbon deposition. The VLS growth process is enabled by molten Al nanoparticles (melting at 660°C), which react with carbon‐nitrogen species from melamine pyrolysis at 1200°C to form Al_4_C_3_ (confirmed by XRD, Figure 2a, and HRTEM lattice fringe at 0.237 nm, Figure 1j). As shown in TEM (Figure 1f) and elemental mapping (Figure 1k), Al_4_C_3_ nanoparticles are encapsulated within carbon layers at the nodes of the bamboo structure. The growth follows a cyclic ‘deposition–migration–extrusion’ mechanism: (i) Carbon‐nitrogen species decompose on the surface of Al_4_C_3_, depositing graphitic carbon (lattice spacing 0.335 nm, Figure 1j); (ii) once the carbon shell reaches a critical thickness (∼10 nm, measured from Figure 1f), the catalyst becomes temporarily encapsulated; (iii) driven by surface tension and continued carbon diffusion, the Al_4_C_3_ particle migrates forward and extrudes from the closed end; (iv) a fresh catalytic surface is exposed, initiating a new growth cycle. This periodic encapsulation and re‐emergence produces uniformly spaced compartments with an internode spacing of 300 ± 20 nm (Figure 1b).

Furthermore, partial carbon layers close before the particles fully separate, thereby trapping the catalyst particles within the central region of the nanotube structure (Figure S2e). Concurrently, EDS mapping and HRTEM confirm that a portion of aluminum reacts with carbon‐nitrogen species to form Al_4_C_3_. Overall, the highly ordered structure of BNCFs is governed by the diffusion behavior of carbon in Al_4_C, catalyst morphological evolution, elemental precipitation, liquid‐solid interfacial tension effects, and particle migration dynamics. Driven by the VLS mechanism, the fibers undergo continuous axial growth, resulting in large aspect ratios and spontaneous intertwining to form a continuous, flexible, and free‐standing film. As shown in Figure S5, the 12 mm freestanding electrode film sustains satisfactory mechanical integrity after folding.

XRD analysis is used to explore the crystalline structures of BNCFs‐Al_4_C_3_, BNCFs, BNCFs/S, and BNCFs/S/PANI composites. The XRD pattern of BNCFs‐Al_4_C_3_ exhibits a broad peak at approximately 26° corresponding to amorphous carbon (Figure 2a), along with separate diffraction peaks that can be attributed to the rhombohedral phase of Al_4_C_3_ (JCPDS No. 50–0740) [40]. Following acid treatment, only the characteristic peaks of graphitic carbon (JCPDS No. 75–2078) remain, indicating the complete elimination of Al_4_C_3_. After sulfur loading, the BNCFs/S composite shows additional diffraction peaks matching the orthorhombic sulfur phase, indicating sulfur impregnation (Figure 2b). The BNCFs/S/PANI composite maintains similar diffraction patterns to BNCFs/S, suggesting the formation of a thin polyaniline coating that preserves electrolyte accessibility while not appreciably affecting the crystalline structure.

Fourier transform infrared spectroscopy (FT‐IR) analysis (Figure 2c) shows distinct absorption bands for BNCFs, PANI, and BNCFs/PANI. The broad peak at 3438 cm^−1^ corresponds to O–H stretching vibrations. In pure BNCFs, the peak at 1632 cm^−1^ corresponds to the stretching vibration of C═O bonds, while the peaks at 1570 and 1211 cm^−1^ correspond to the stretching vibrations of C═C and C─O bonds, respectively [41]. The FT‐IR spectra of PANI and BNCFs/PANI show peaks at 1564 and 1480 cm^−1^, which correspond to the stretching vibrations of C═N and C═C bonds in quinoid and benzenoid rings of PANI [42]. The absorption peak at 1300 cm^−1^ coincides with the C─N stretching vibration of aromatic amines [43]. The peaks at 1133 and 795 cm^−1^ belong to the stretching vibrations of protonated imine groups (–NH^+^ =) and aromatic C─H bonds, respectively [44]. In the Raman spectra of BNCFs‐Al_4_C_3_ and BNCFs (Figure 2d), the unique peaks at 1346 (D‐band) and 1589 cm^−1^ (G‐band) correspond to the disordered form of amorphous carbon and the graphitic structure, respectively [17]. The ratio of the peak intensities (I_D_/I_G_) serves to determine the graphitization degree of the material. A higher graphitization degree facilitates enhanced electronic conductivity, thereby intensifying sulfur consumption during charge/discharge processes [45]. After acid treatment, the surface defect density of BNCFs increases significantly, furnishing greater connection sites for electrolyte ions [46]. In the Raman spectra of the BNCFs/S and BNCFs/S/PANI composites (Figure S6a), the three characteristic peaks at 162, 229, and 482 cm^−1^ are assigned to sulfur, signaling successful loading of sulfur [47]. Voigt function fitting analysis reveals that the sp^2^/sp^3^ ratio of BNCFs/S/PANI (1.41) is higher than that of BNCFs/S (1.16), indicating that PANI incorporation further enhances the graphitization degree of the material (Figure S6b,c). This improvement enhances the overall conductivity of the composite, thereby facilitating efficient electron transport during charge/discharge cycles [48].

The sulfur contents of BNCFs/S and BNCFs/S/PANI were determined by thermogravimetric analysis (TGA) under a nitrogen atmosphere (Figure 2e). Both BNCFs/S and BNCFs/S/PANI materials exhibits considerable weight loss between 105°C–250°C, with estimated sulfur contents of 61.7% and 61.4%, respectively. The high sulfur loading contributes to enhancing the energy density of Li–S batteries. The contents of C, O, and N in the BNCFs/PANI were determined by X‐ray photoelectron spectroscopy (XPS) to be approximately 86.11%, 9.81%, and 4.08%, respectively (Table S1). The specific surface area and pore geometry of BNCFs‐Al_4_C_3_ and BNCFs were examined using N_2_ adsorption‐desorption isotherms and pore size variation evaluation. Type IV isotherm curves were observed for both materials (Figure 2f). The specific surface area of BNCFs‐Al_4_C_3_ was 21.4 m^2^ g^−1^, while that of BNCFs after acid treatment increased to 35.6 m^2^ g^−1^.

The higher specific surface area can provide more space for sulfur loading and plentiful catalytic sites, thereby suppressing the dissolution of LiPSs. Similarly, BNCFs‐Al_4_C_3_ and BNCFs exhibited a narrow pore size distribution of 0.7–5 nm, indicating the presence of micropores and mesopores, which allow for efficient infiltration and optimizing the kinetics of LiPSs redox reactions [49]. The specific surface areas of BNCFs/S and BNCFs/S/PANI were 11.2 and 12.6 m^2^ g^−1^, respectively, implying that sulfur was almost entirely disseminated within the pores (Figure S6d). Meanwhile, the BNCFs/S/PANI membrane offers multiple benefits, such as sufficient internal cavities to isolate sulfur species and continuous open channels that facilitate accelerated ion transport in the electrolyte, all within a single host structure.

XPS analysis reveals that BNCFs‐Al_4_C_3_ is mainly consists of C, N, Al, and O elements, while the Al signal disappears in BNCFs after acid treatment (Figure S7a,d). The C 1s spectrum of BNCFs‐Al_4_C_3_ was fitted to three peaks related to C─C/C═C (283.6 eV), C─N (∼284.6 eV), and C═O (287.8 eV) (Figure S7b) [50]. Particularly, the C 1s spectrum of acid‐treated BNCFs, two different bands appear at ∼285.8 and 291.0 eV, which are assigned to C─O and O─C═O bonds, respectively (Figure S7e). The observed peak shifts show an increase in oxygen‐containing functional groups due to the reaction with acid [51]. The fitted N 1s spectra of BNCFs‐Al_4_C_3_ and BNCFs exhibit four distinct peaks corresponding to pyridinic N, pyrrolic N, graphitic N, and oxidized N (Figure S7c,f) [52]. The full XPS spectra of BNCFs/S/PANI showed characteristic peaks at 168.0 eV (S 2p), 284.9 eV (C 1s), 400.1 eV (N 1s), and 531.6 eV (O 1s) (Figure S8a). The spectrum of C 1s in BNCFs/S/PANI was fitted to five peaks representing C─C/C═C, C─N, C─O, C═O, and O─C═O bonds (Figure 2g). The appearance of C─N bonds verify the presence of nitrogen‐containing functional groups. The C═O bonds may appear during oxidative stabilization process. The N 1s spectrum (Figure 2h) exhibits five distinct peaks at 399.2 eV (pyridinic N/–N =), 400.0 eV (–N–), 400.7 eV (pyrrolic N), 401.4 eV (graphitic N/–NH^+^ =), and 405.1 eV (oxidized N), indicating the successful in situ growth of polyaniline on nitrogen‐doped graphitic carbon [39]. The lone electron pairs and high electronegativity of pyridinic N and pyrrolic N can efficiently anchor Li^+^ in LiPSs through LiS*_n_* Li^+^–N bonding, crucially mitigating the shuttle effect [53]. The S 2p spectra (Figure 2i) were fitted to S 2p_3/2_ (163.6 eV), S 2p_1/2_ (164.6 eV), sulfate (168.3 eV), and S─C bonds (169.3 eV) [54]. In O 1s spectrum (Figure S8b), the wavelength at 531.6 eV is imputed to hydroxyl groups, while the peak at 533.1 eV relates to C═O bonds [55].

### 2.2 Electrochemical Performance

**FIGURE 3:**
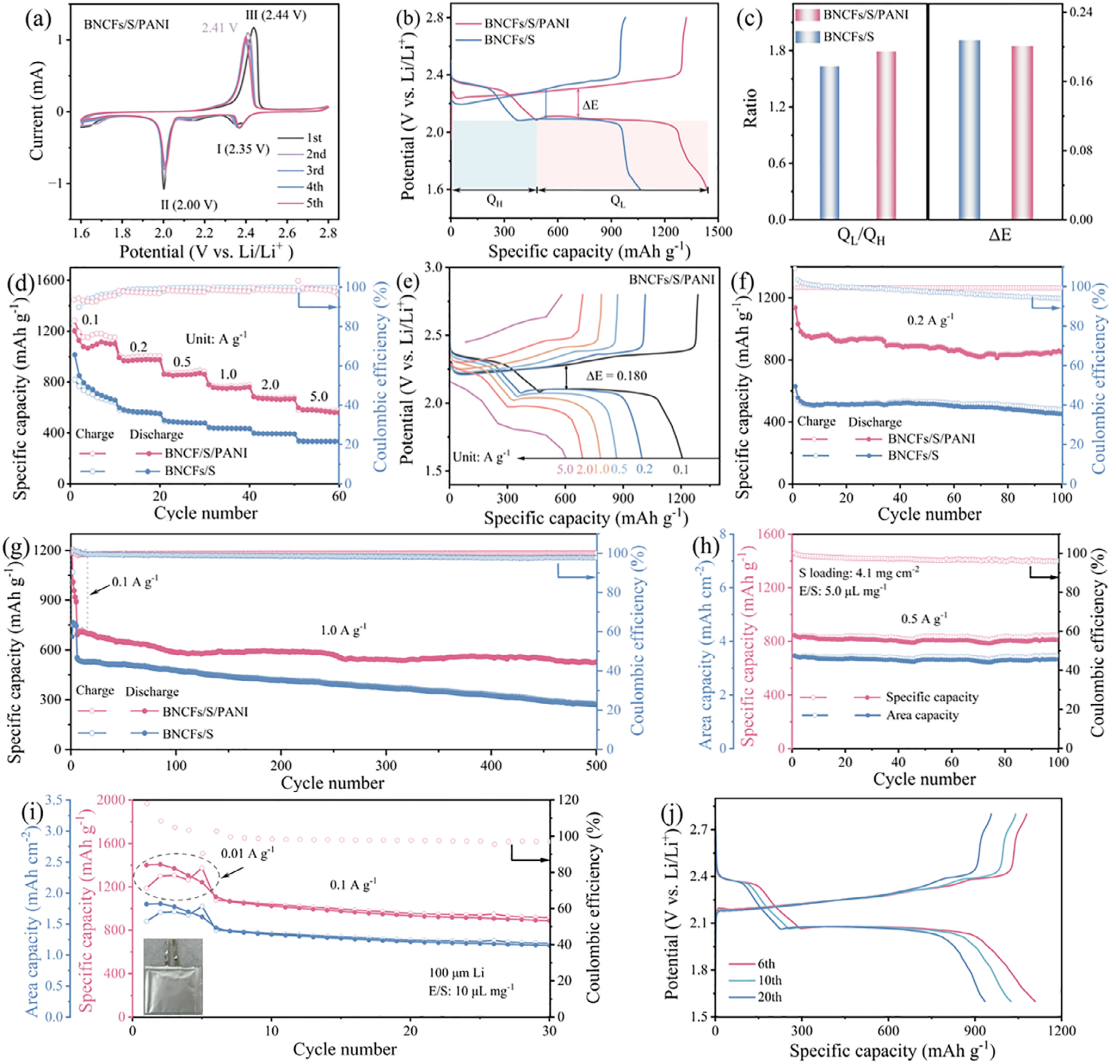
(a) Initial five cycles CV curves of BNCFs/S/PANI at a scan rate of 0.1 mV s^−1^. (b) Galvanostatic charge‐discharge curves at 0.1 A g^−1^ of BNCFs/S/PANI and BNCFs/S. (c) The calculated capacities of the low discharge plateau (Q_L_) value/ high discharge plateau (Q_H_); Voltage polarization between discharge and charge curves. (d) Rate performance of BNCFs/S/PANI and BNCFs/S. (e) Galvanostatic charge/discharge profiles of BNCFs/S/PANI. Cycling performance of BNCFs/S/PANI and BNCFs/S at (f) 0.2 A g^−1^ and (g) 1.0 A g^−1^. (h) Cycling performance of BNCFs/S/PANI with high S loadings at 0.5 A g^−1^. (i) Cycling performance of the pouch cell using BNCFs/S/PANI at 0.1 A g^−1^. (j) The charge/discharge curves of the pouch cell at 0.1 A g^−1^.

To assess the electrochemical properties of the self‐supporting BNCFs/S/PANI cathode, the electrochemical performance of both BNCFs/S and BNCFs/S/PANI was tested. The first five CV curves of self‐supporting BNCFs/S/PANI cathode are shown in Figure 3a, recorded at a scan rate of 0.1 mV s^−1^ within a voltage range of 1.6–2.8 V. The reduction peaks at 2.35 and 2.00 V correspond to the two‐step reduction process of S_8_ to soluble long‐chain polysulfides (Li_2_S*_n_*, 4 ≤ *n* ≤ 8) and followed by the conversion of long‐chain Li_2_S*_n_* to insoluble short‐chain Li_2_S/Li_2_S_2_, similarly [56]. The oxidation peak at 2.44 V corresponds to the reversal process, where Li_2_S/Li_2_S_2_ is oxidized to long‐chain Li_2_S*_n_* and ultimately to S_8_ [57]. Notably, the subsequent four cycles exhibit well‐overlapped redox peaks, suggesting excellent cycling stability and electrochemical flexibility of the BNCFs/S/PANI cathode. Conversely, the CV curves of the self‐supporting BNCFs/S cathode are presented (Figure S9a). Compared to BNCFs/S/PANI, BNCFs/S possesses wider redox peak potential gaps, lower peak currents, and poorer overlap, which can be ascribed to its weaker adsorption potential for LiPSs and higher polarization due to frequent polysulfide shuttling, resulting in lesser sulfur utilization. The discharge/charge profiles of BNCFs/S/PANI and BNCFs/S cathodes at 0.1 A g^−1^ (Figure 3b) constitute two discharge depressions and one charge depression, consistent with the CV results. The BNCFs/S/PANI cathode delivers a higher initial discharge specific capacity (1436.1 mAh g^−1^) compared to BNCFs/S. Q_H_ presents the unique discharge capacity for the turning of S_8_ to liquid Li_2_S_4_, while Q_L_ corresponds to the capacity for the conversion of Li_2_S_4_ to soluble Li_2_S. As shown in Figure 3c, the higher Q_L_/Q_H_ ratio (1.78) for BNCFs/S/PANI compared to BNCFs/S (1.63), along with a lower polarization voltage (Δ*E* = 0.201 V), indicates more robust catalytic activity. These findings indicate that BNCFs/S/PANI exhibits improved electrochemical kinetics, benefiting from the conductive PANI coating and its redox activity, which collectively contribute to reduced polarization during cycling [39].

To evaluate the electrochemical performance of BNCFs/S and BNCFs/S/PANI under various current densities, rate capability tests were conducted (Figure 3d). At 0.1 A g^−1^, the BNCFs/S/PANI cathode delivered an initial discharge specific capacity of 1205.6 mAh g^−1^, significantly higher than that of the BNCFs/S cathode. Among them, the phenomenon of capacity initially decreasing and then increasing for the BNCFs/S/PANI cathode at 0.1 A g^−1^ is attributed to insufficient electrolyte wetting at the initial stage, which causes an initial capacity decrease, followed by a gradual capacity recovery as the wetting becomes sufficient. As the current density increased to 0.2, 0.5, 1.0, 2.0, and 5.0 A g^−1^, the BNCFs/S/PANI delivered discharge capacities of 996.5, 865.5, 782.2, 689.0, and 601.9 mAh g^−1^, respectively. Furthermore, the rate performance of BNCFs/S/PANI at different aniline dosages is presented in Figure S9b, where the 15 µL dosage demonstrates the best performance. By contrast, the BNCFs/S cathode shows much lower capacities across all current densities, delivering only 1015.8, 597.6, 499.6, 445.0, 399.9, and 339.3 mAh g^−1^ at the relevant current densities. The elevated rate performance of BNCFs/S/PANI can be attributed to its unique hollow bamboo‐like carbon nanofiber structure combined with the PANI coating, which effectively accelerates Li^+^ transport kinetics. Moreover, the oxygen‐doped and N‐doped carbon nanotubes exhibit both physical and chemical affinity towards sulfur and lithium sulfide species, significantly enhancing charge transfer dynamics and enabling excellent cycling stability even at high current density.

The electrochemical charge/discharge performance of BNCFs/S and BNCFs/S/PANI was evaluated at various current densities (Figure 3e and Figure S9c). For instance, the BNCFs/S/PANI cathode retains two distinct discharge peaks even at high current density. At 0.1 A g^−1^, BNCFs/S/PANI exhibited the lowest polarization (Δ*E* = 0.180 V), indicating enhanced reaction kinetics and faster polysulfide redox kinetics than BNCFs/S. The cycling performance of both cathodes at 0.2 A g^−1^ are analyzed in Figure 3f. The BNCFs/S/PANI cathode delivered an initial discharge capacity of 1135.5 mAh g^−1^ and retained 852.4 mAh g^−1^ after 100 cycles, achieving 75.1% capacity retention. Comparatively, BNCFs/S retained only 71.7% of its initial capacity after 100 cycles under identical conditions. The BNCFs/S/PANI cathode also demonstrated excellent cycling endurance at 1.0 A g^−1^ (Figure 3g), delivering an initial discharge capacity of 691.2 mAh g^−1^ and retaining 527.1 mAh g^−1^ over 500 cycles, with a minimal capacity decay rate of 0.047% per cycle. In contrast, the BNCFs/S cathode exhibits a lower initial discharge specific capacity of 582.8 mAh g^−1^, which further decreases to only 297.4 mAh g^−1^ after 500 cycles. At elevated current densities of 2.0 and 5.0 A g^−1^ (Figure S9d,e), the BNCFs/S/PANI cathode maintained initial discharge specific capacities of 522.1 and 337.9 mAh g^−1^, with a 0.096% and 0.109% capacity decay rate per cycle after 500 cycles, respectively. The above results indicate that the highly conductive carbon film and the well‐dispersed sulfur within the hierarchical structure facilitate redox reactions and sulfur confinement. Meanwhile, the BNCFs not only provide abundant pathways but also promote electrolyte transport. Accordingly, the BNCFs/S/PANI cathode indicates exceptional electrochemical performance.

The sulfur loading of active materials and the practical electrolyte/sulfur (E/S) ratio are key criteria for evaluating the practicality of Li–S batteries. The cycling performance of the BNCFs/S/PANI cathode was evaluated under challenging circumstances, including a high sulfur loading of 4.1 mg cm^−2^ and a lean electrolyte condition (E/S ratio of ∼5 µL mg^−1^), as shown in Figure 3h. The initial discharge specific capacity at 0.5 A g^−1^ was 845.1 mAh g^−1^, equivalent to an areal capacity of 3.46 mAh cm^−2^. After 100 cycles, the battery retained a specific capacity of 811.6 mAh g^−1^ (3.32 mAh cm^−2^). In contrast, as shown in Figure S10a, the BNCFs/S cathode (without PANI) retained only 416.8 mAh g^−1^ (1.71 mAh cm^−2^) after 100 cycles under the same conditions. Furthermore, a pouch cell was assembled using the BNCFs/S/PANI cathode with a sulfur loading of 1.3 mg cm^−2^ and an E/S ratio of 10 µL mg^−1^ (Figure S11), and its cycling performance was determined. The BNCFs/S/PANI cathode delivered an outstandingly high initial discharge specific capacity of 1402.5 mAh g^−1^ at 0.01 A g^−1^ (Figure 3i). Even when the current density was increased to 0.1 A g^−1^, the material retained a high discharge specific capacity of 1108.3 mAh g^−1^. After 25 cycles, the specific capacity stabilized at 887.8 mAh g^−1^ (1.15 mAh cm^−2^). For comparison, as shown in Figure S10b, after 25 cycles under the same conditions, the capacity of the BNCFs/S cathode dropped to 98.5 mAh g^−1^ (0.13 mAh cm^−2^). Based on the total mass of the BNCFs/S/PANI pouch cell, the corresponding energy density was calculated to be approximately 67.8 Wh kg^−1^. The relatively moderate value is mainly attributed to the low sulfur loading employed in the present pouch cell. Nevertheless, the freestanding BNCFs/S/PANI cathode exhibits considerable potential for practical flexible and lightweight Li–S pouch cells, owing to its binder‐free conductive framework and efficient sulfur utilization. Additionally, the discharge/charge curves at 0.1 A g^−1^ exhibited profiles similar to those of coin cells (Figure 3j). These results illustrate that the BNCFs/S/PANI pouch cell also achieves excellent performance. Table S2 compares the cycling performance of the BNCFs/S/PANI cathode with various self‐supporting sulfur host materials (such as WLC‐CNT [36], iPANI@rGO‐CNTs [39], High O_x_‐SWCNTs [58], S@CCG [59], P‐Mn_3_O_4−x_ [60], and other carbon‐based cathodes [61, 62, 63, 64]. The BNCFs/S/PANI‐based battery exhibits superior electrochemical performance.

**FIGURE 4:**
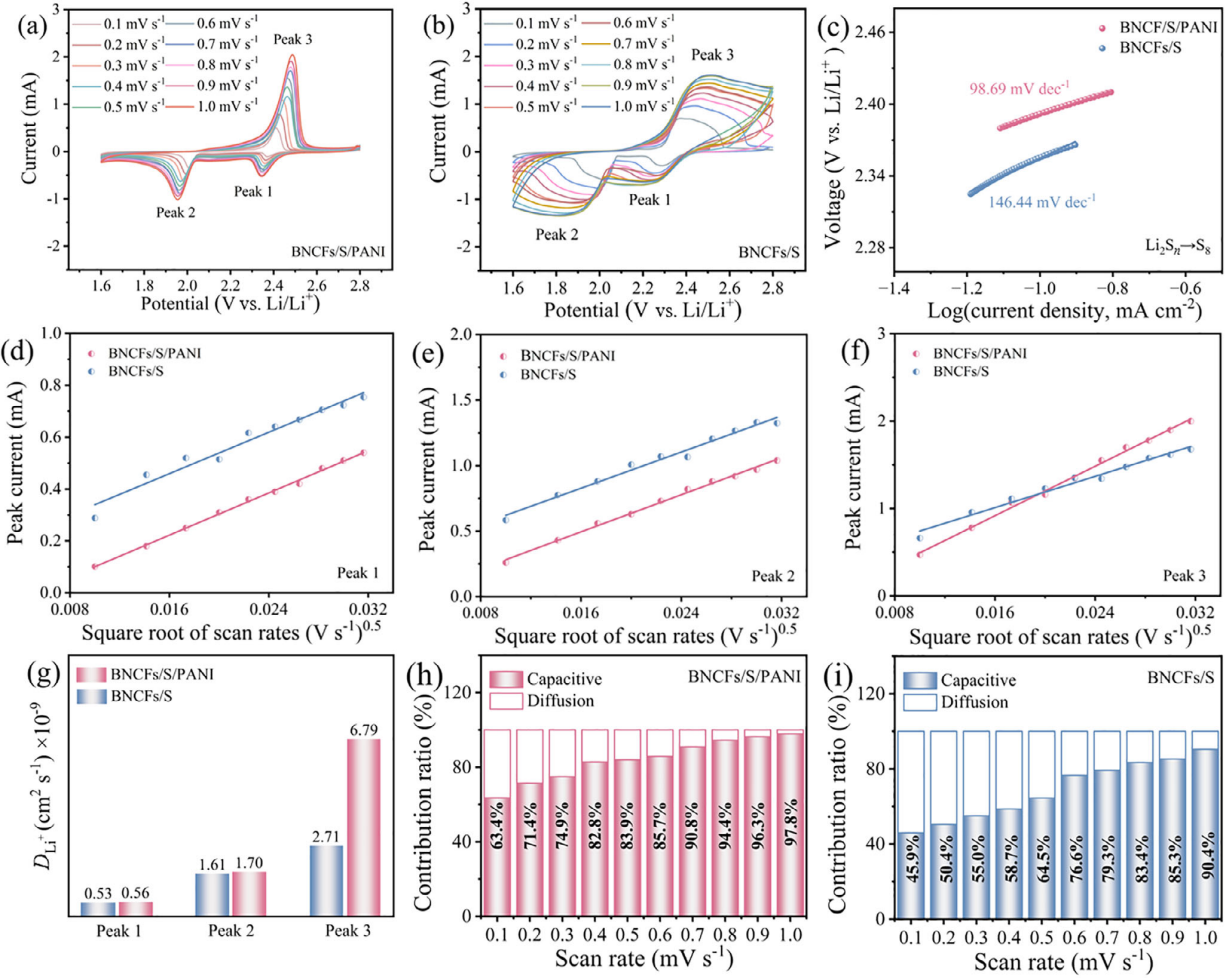
CV curves of (a) BNCFs/S/PANI and (b) BNCFs/S cathodes at various scan rates. (c) Tafel plots derived from Peaks 3. (d) First cathodic reduction process (peak 1: S/Li_2_S*_n_*). (e) Second cathodic reduction process (peak 2: Li_2_S*_n_*/Li_2_S_2_/Li_2_S). (f) Anodic oxidation process (peak 3: Li_2_S/Li_2_S*_n_*) versus the square root of the scan rates. (g) Li^+^ diffusion coefficient of BNCFs/S/PANI and BNCFs/S. Column diagrams of the capacitive ratio for (h) BNCFs/S/PANI and (i) BNCFs/S at various scan rates.

To investigate the effect of Li^+^ diffusion rates in the reaction kinetics of polysulfide conversion, CV measurements of BNCFs/S and BNCFs/S/PANI were conducted at scan rates ranging from 0.1 to 1.0 mV s^−1^ (Figure 4a,b). Tafel plots derived from the reduction peak (Peak 2) and oxidation peak (Peak 3) in the CV curves at 0.1 mV s^−1^ (Figure 4c and Figure S12) were used to evaluate the effect of electrocatalysis on charge transfer kinetics. In comparison to BNCFs/S (reduction peak: 44.48 mV dec^−1^, oxidation peak: 146.44 mV dec^−1^), BNCFs/S/PANI exhibited lower Tafel slopes (reduction peak: 36.81 mV dec^−1^, oxidation peak: 98.69 mV dec^−1^), confirming its enhanced ability to accelerate the reaction kinetics of LiPSs conversion. The Randles‐Sevcik equation states that, the peak current (*i*) is linearly proportional to the square root of scan rate (*v*) (Figure 4a,b): $I_{p}=2.69\times 10^{5}n^{1.5}SD_{Li^{+}}^{0.5}C_{Li^{+}}v^{0.5}$. *I_p_*, *n*, *S*, $D_{Li^{+}},\;C_{Li^{+}}$, and *v* represent peak current, number of electron transfer, electrode area, Li^+^ diffusion coefficient, Li^+^ concentration, and scan rate, respectively. Therefore, the slope is proportional to the Li^+^ diffusion rate [57]. After fitting the relationship between *I_p_* and *v* ^0.5^, BNCFs/S/PANI exhibited steeper slopes for both oxidation and reduction activities (Figure 4d–f). The estimated Li^+^ diffusion coefficients (Figure 4g) demonstrate that the BNCFs/S/PANI cathode attains the highest diffusion coefficients during redox reactions compared to BNCFs/S, indicating rapid Li^+^ transfer kinetics. This ensures the superior electrochemical performance of the BNCFs/S/PANI cathode at high rates. Meanwhile, *i* shows a typical dependence on *v*, which can be mathematically described by: *i = k*_1_ *v + k*_2_ *v* ^1/2^ *= av^b^* and *log(i) = blog(v) + log(a)*, where *a* and *b* are constants, with the terms *k*_1_ *v* and *k*_2_ *v* ^1/2^ harmonious to capacitive and diffusion‐controlled contributions, respectively [27]. As evidenced in Figure 4h,i, the capacitive contribution increases progressively with scan rate for both BNCFs/S and BNCFs/S/PANI composites, reaching 97.8% for BNCFs/S/PANI at 1.0 mV s^−1^. These results indicate that the PANI coating enhances both Li^+^ diffusion and the conversion kinetics of LiPSs.

**FIGURE 5:**
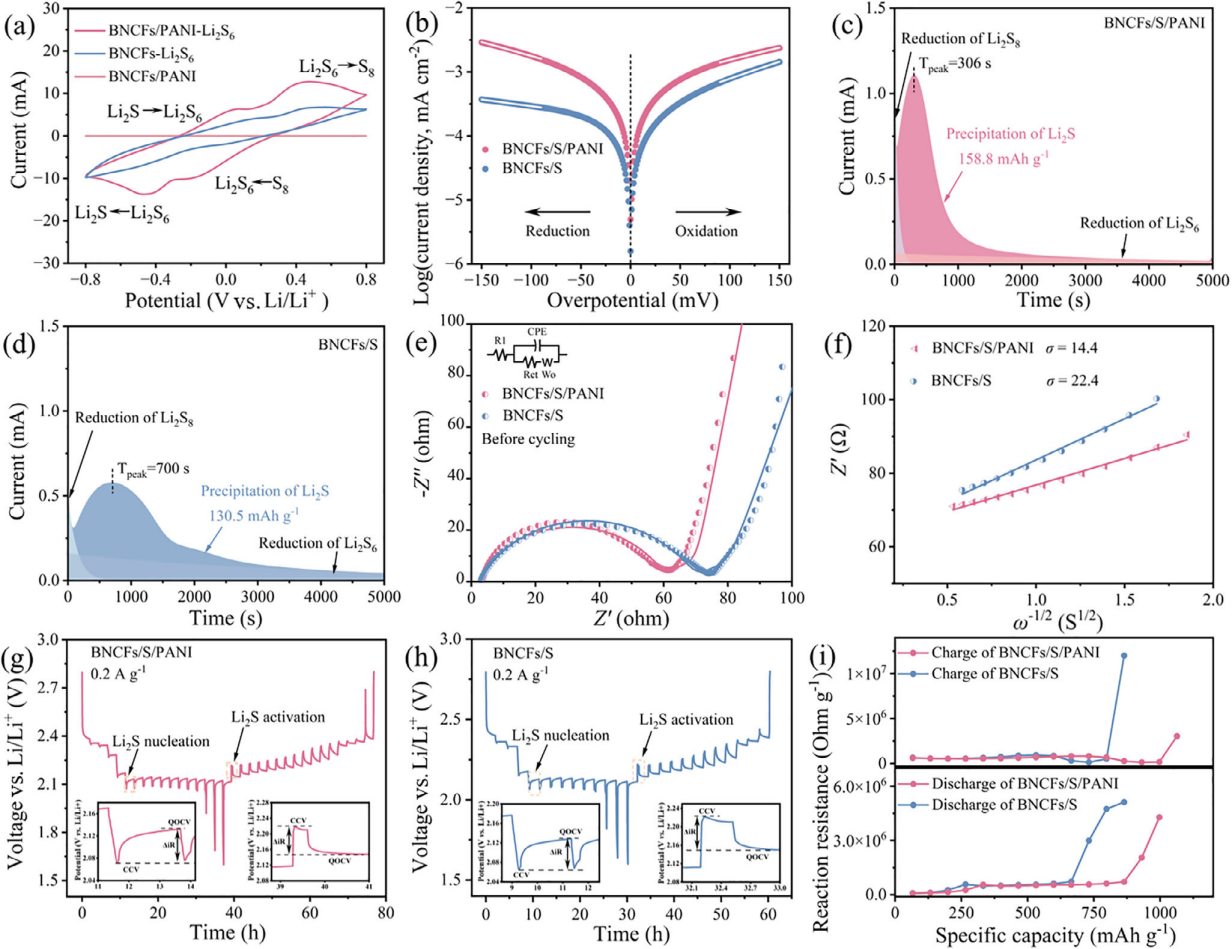
(a) CV curves of Li_2_S_6_ symmetric cells with various electrodes (BNCFs/PANI and BNCFs) and Li_2_S_6_−containing electrolyte. (b) The Tafel plots for various catalysts, and the linear portion of the fit, calculate the corresponding exchange current density. Potentiostatic nucleation curves of Li_2_S with (c) BNCFs/PANI, and (d) BNCFs. (e) Nyquist plot of BNCFs/S/PANI and BNCFs/S before cycling test. (f) Plot of the real part of impedance (*Z'*) as a function of the inverse square root of angular frequency (*ω* ^−1/2^). GITT time‐potential distribution of (g) BNCFs/S/PANI and (h) BNCFs/S in discharge/charge process. (i) In situ reaction impedances of BNCFs/S/PANI and BNCFs/S during discharge and charge.

To investigate the dynamic adsorption behavior and intrinsic catalytic activity of BNCFs and BNCFs/PANI in LiPSs conversion processes, symmetric cells were assembled using Li_2_S_6_ electrolyte and symmetric electrodes, followed by CV measurements within a potential window of −0.8 to 0.8 V (vs. S/S^2−^). As shown in Figure 5a, compared with BNCFs, the BNCFs/PANI electrode demonstrates prominent redox peaks, enhanced current response, and lower polarization voltage, indicating significantly improved electrochemical reversibility and reaction kinetics of LiPSs mediated BNCFs/PANI mediation. Furthermore, Tafel analysis was conducted to evaluate the LiPSs conversion efficiency of BNCFs and BNCFs/PANI. In Figure 5b, the BNCFs/PANI electrode shows superior exchange current densities (*i*_0_) of 0.65 mA cm^−2^ (reduction) and 0.63 mA cm^−2^ (oxidation) at 2 mV s^−1^, markedly higher than those of BNCFs (0.18 mA cm^−2^ for reduction and 0.23 mA cm^−2^ for oxidation). These results provide definitive evidence for the enhanced LiPSs conversion capability enabled by the PANI−modified architecture. The catalytic kinetics of LiPSs conversion to Li_2_S on BNCFs/PANI were investigated through Li_2_S nucleation experiments. As shown in Figures 5c and d, the current response times for the conversion of LiPSs to Li_2_S on BNCFs/PANI and BNCFs at 2.05 V were 306 and 700 s, respectively, indicating faster Li_2_S nucleation on BNCFs/PANI. In addition, the BNCFs/PANI electrode delivered a higher Li_2_S nucleation capacity (158.8 mAh g^−1^) compared to BNCFs (130.5 mAh g^−1^). This enhancement originates from the reversible electron rearrangement of −NH═groups in PANI, which efficiently modulates LiPSs conversion behavior and promotes sulfur utilization.

EIS measurements were performed on both BNCFs/S and BNCFs/S/PANI electrodes prior to cycling, with the resultant spectra comprising characteristic charge transfer resistance (*R*_ct_) and Warburg impedance (*W*_o_), as illustrated in Figure 5e. Compared to BNCFs/S, BNCFs/S/PANI exhibits the smallest *R*_ct_ (56.2 Ω) and *W*_o_ (31.2 Ω). The linear relationship between the real part of impedance (*Z*’) and the square root of angular frequency (*ω* ^−1/2^) for the battery before cycling is presented in Figure 5f. The analysis was primarily based on the equations 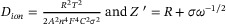, where *R*, *T*, *A, n*, *F, C*, and *σ* represent the gas constant, absolute temperature, electrode area, number of electrons transferred, Faraday constant, ion concentration, and Warburg coefficient, respectively [27]. The BNCFs/S/PANI composite shows significantly enhanced Li^+^ diffusion coefficients (Table S3), indicating superior electrical conductivity and ion transport capability. EIS after 500 cycles (Figure S13) revealed an additional semicircle for both BNCFs/S and BNCFs/S/PANI, corresponding to the interfacial contact resistance (*R*_f_). Meanwhile, both systems exhibit reduced *R*_ct_, with BNCFs/S/PANI showing the lowest values (Table S4). These results demonstrate that the BNCFs provide a highly conductive framework, while PANI further enhances interfacial charge transfer via its intrinsic conductivity, synergistically reducing the internal resistance of the electrode.

To further demonstrate the mechanical flexibility and structural stability of the prepared self‐supporting electrode, EIS measurements of the assembled pouch cell were conducted before and after folding. As shown in Figure S14 and Table S5, the BNCFs/S/PANI electrode exhibits only a negligible change in impedance after folding, indicating that its conductive network and overall electrode structure remain well‐integrated during mechanical deformation. This demonstrates the excellent mechanical flexibility and stable electron/ion transport capability of the self‐supporting electrode. Notably, a slight decrease in impedance was observed after folding, which may be attributed to the closer interfacial contact among the cell components under bending conditions, thereby facilitating charge transfer.

The electrochemical impedance evolution during charge/discharge processes was monitored in situ using the galvanostatic intermittent titration technique (GITT) at a constant current density of 0.2 A g^−1^ (Figure 5g,h). The internal battery resistance (Δ*R*) was calculated from the applied current and the voltage difference between closed−circuit voltage (CCV) and quasi−open−circuit voltage (QOCV), using the following formula under equal mass loading conditions: Δ𝑅_internal_(Ω) = |Δ𝑉_QOCV_−Δ𝑉_CCV_ |/(*I*_applied_ × *M* ^2^_mass loading_) [65]. The in situ resistance profiles during cycling are shown in Figure 5i, revealing that the BNCFs/S cathode exhibits higher resistance than the BNCFs/S/PANI counterpart, indicating that the smaller internal resistance of the BNCFs/S/PANI cathode results in a lower energy barrier for the oxidative conversion of Li_2_S, thereby significantly enhancing the electrochemical reaction kinetics. The polyaniline coating catalyzes the redox conversion of sulfur species and lowers the reaction energy barrier, resulting in higher conductivity and a lower energy barrier for interfacial charge transfer kinetics for BNCFs/S/PANI cathodes [39]. In addition, Li^+^ can be rapidly transferred through the hollow carbon fiber conductive network system to swiftly participate in the redox reaction.

**FIGURE 6:**
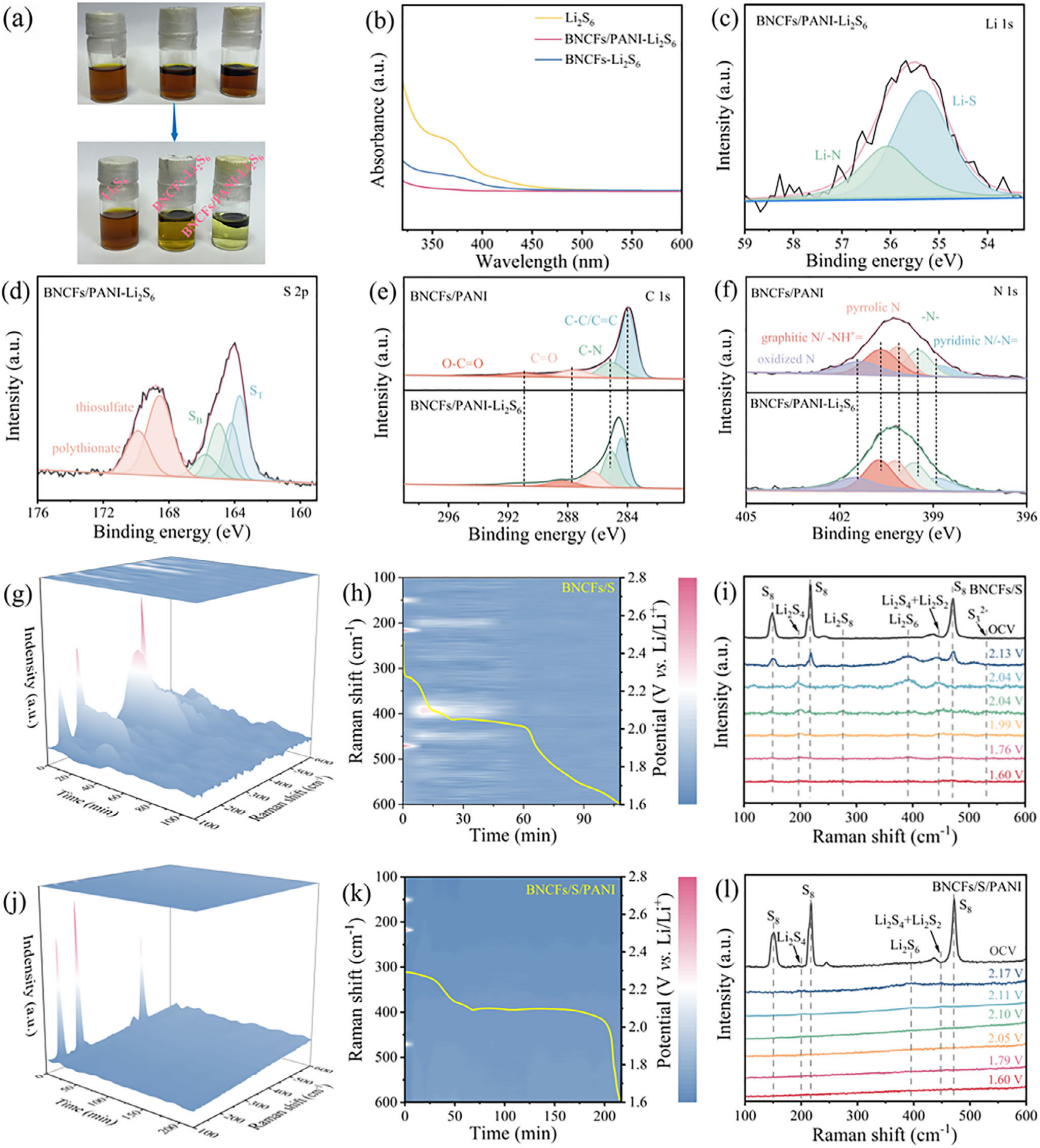
(a) Optical photographs of sealed vials of a Li_2_S_6_/DOL/DME solution contacted with BNCFs /PANI and BNCFs after 10 h. (b) UV‐vis spectra of the residual solution after adsorption for 10 h. (c) Li 1s XPS spectrum of BNCFs/PANI‐Li_2_S_6_. (d) S 2p XPS spectrum of BNCFs/PANI‐Li_2_S_6_. (e) C 1s XPS spectra of BNCFs/PANI‐Li_2_S_6_ and BNCFs/PANI. (f) N 1s XPS spectra of BNCFs/PANI‐Li_2_S_6_ and BNCFs/PANI. The three‐dimensional (3‐D) and two‐dimensional (2‐D) time‐resolved Raman spectra of (g) BNCFs/S and (j) BNCFs/S/PANI. During the discharge process, in situ time‐resolved Raman spectra of (h) BNCFs/S and (k) BNCFs/S/PANI. Corresponding LiPSs Raman signals of (i) BNCFs/S and (l) BNCFs/S/PANI collected during discharge.

The adsorption capability of the cathode material for LiPSs plays a crucial role in suppressing the shuttle effect. Therefore, visual adsorption experiments and UV‐vis spectroscopy analysis were conducted in a 0.1 mol L^−1^ Li_2_S_6_ solution. As shown in Figure 6a, after standing for 10 h, the originally dark yellow solution containing BNCFs/PANI became nearly colorless compared to the solution with BNCFs alone. The Li_2_S_6_ solution after adsorption was further tested using UV‐vis absorption spectroscopy (Figure 6b). The absorption peaks of the BNCFs/PANI‐Li_2_S_6_ solution were the weakest, indicating that the in situ grown PANI on BNCFs exhibits strong adsorption capability for Li_2_S_6_. The chemical interaction between BNCFs/PANI and LiPSs was further inspected through XPS analysis. The full XPS spectrum of BNCFs/PANI‐Li_2_S_6_ exhibited distinct Li 1s and S 2p peaks, verifying the interaction between BNCFs/PANI and Li_2_S_6_ (Figure S15). In the Li 1s spectrum (Figure 6c), the peaks at 55.38 and 56.08 eV were assigned to Li–S and Li–N bonds, respectively, demonstrating strong chemical interactions between BNCFs/PANI and Li_2_S_6_ [66]. The S 2p spectrum (Figure 6d) was deconvoluted into four bands, corresponding to terminal sulfur (S_T_), bridging sulfur (S_B_), thiosulfate and polysulfates [67]. In the C 1s and N 1s spectra (Figure 6e,f), the peak of BNCFs/PANI‐Li_2_S_6_ exhibited a positive shift compared to those of pristine BNCFs/PANI, confirming electron transfer between BNCFs/PANI and Li_2_S_6_ [68].

Further in situ Raman spectroscopy was utilized to analyze the real‐time conversion of LiPSs by BNCFs/S and BNCFs/S/PANI composites (Figure 6g–l). The 3‐D and 2D time‐resolved Raman spectra (Figure 6g,h,j,k) revealed that almost no signals of sulfur‐containing species were detected throughout the discharge process in batteries with BNCFs/S/PANI cathodes. For both BNCFs/S/PANI and BNCFs/S cathodes during discharge (Figure 6i,l), only the characteristic peaks of S_8_ (150, 216, and 472 cm^−1^) were observed in the initial stage. Subsequently, as the discharge process proceeded, the S_8_ peaks gradually vanished. Near 2.1 V, the peak at 196.1 cm^−1^ relates to Li_2_S_4_, the peak at 389.5 cm^−1^ primarily corresponds to Li_2_S_6_, and the peak at 449.0 cm^−1^ due to Li_2_S_4_ and Li_2_S_2_, which is attributed to the conversion of S_8_ to lower‐order polysulfides during discharge [8]. For the BNCFs/S cathode, the peaks of Li_2_S_6_, Li_2_S_4_, and Li_2_S_2_ remained detectable until the end of the second plateau. In contrast, the BNCFs/S/PANI cathode exhibited extremely weak common LiPSs peaks in the subsequent discharge process. These findings suggest that BNCFs/S/PANI can effectively promote LiPSs conversion, enhance sulfur utilization, and thereby mitigate the shuttle effect to achieve high stability.

To directly inspect the structural stability of the BNCFs/S/PANI cathode, the battery, cycled for 500 times at 5.0 A g^−1^ was disassembled and analyzed via SEM. The bamboo‐like hollow tubular structure of BNCFs/S/PANI remained intact without collapse, demonstrating its ability to effectively mitigate volume expansion during cycling (Figure S16). These observations support the exceptional structural durability and interconnected framework of BNCFs/S/PANI, which contribute to its remarkable cycling performance. The XRD pattern of the cycled BNCFs/S/PANI is presented in Figure S17. Its characteristic peaks remain consistent with the standard patterns of carbon, demonstrating the high phase stability of the material. This ensures the sustained activity of the BNCFs/PANI. For further affirmation, a battery cycled for 100 times at 0.2 A g^−1^ was disassembled. In comparison to the separator from the BNCFs/S battery, the separator of the BNCFs/S/PANI battery appeared semi‐transparent white (Figure S18), indicating reduced polysulfide shuttling. Moreover, SEM images revealed a smoother lithium metal surface in the BNCFs/S/PANI battery (Figure S19). These conclusions collectively demonstrate that BNCFs/S/PANI successfully reduces LiPSs shuttling, thereby enhancing sulfur usage and overall battery performance.

### 2.3 Density Functional Theory (DFT) Calculations

**FIGURE 7:**
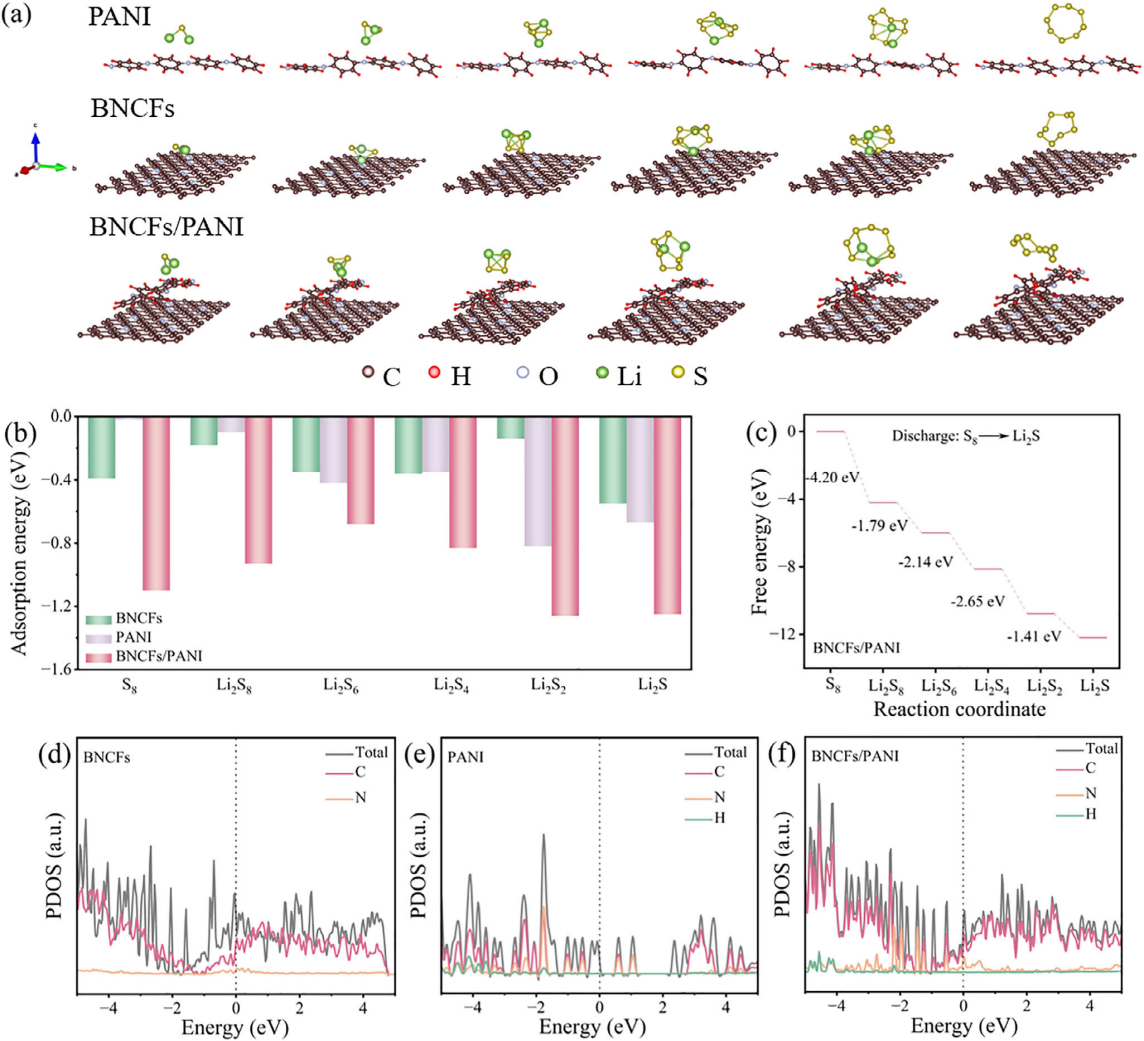
(a) Adsorption models of S_8_ and Li_2_S*_n_* (*n* = 8, 6, 4, 2, 1) in BNCFs, PANI, and BNCFs/PANI. (b) Adsorption energies of S_8_ and Li_2_S*_n_* with BNCFs, PANI, and BNCFs/PANI substrate. (c) Gibbs energy profiles for the reduction process of BNCFs/PANI. PDOS of (d) BNCFs, (e) PANI, and (f) BNCFs/PANI.

Density functional theory (DFT) calculations were conducted to explore the synergistic effects of BNCFs and BNCFs/PANI at the atomic level. The substrate models of S_8_, Li_2_S_8_, Li_2_S_6_, Li_2_S_4_, Li_2_S_2,_ and Li_2_S on BNCFs, PANI, and BNCFs/PANI are displayed in Figure 7a, with the associated adsorption energies shown in Figure 7b. Due to the BNCFs–S and PANI–S interaction effects, the adsorption energies of Li_2_S*_n_* on BNCFs/PANI were found to be higher than those on BNCFs alone. To estimate the catalytic role of BNCFs/PANI in the sulfur conversion process, the Gibbs free energy (ΔG) was computed. The adsorption energies of S_8_, Li_2_S_8_, Li_2_S_6_, Li_2_S_4_, Li_2_S_2_ and Li_2_S on BNCFs/PANI are presented in Figure 7c. In the reduction process from solid S_8_ to solid Li_2_S, the conversion of Li_2_S_2_ to Li_2_S exhibits the lowest ΔG barrier, which serves as the rate‐determining step (RDS) for the overall discharge process. Finally, the projected density of states of Li_2_S_6_ on several substrates were calculated to confirm the interaction between Li_2_S_6_ and BNCFs/PANI (Figure 7d–f). Compared to BNCFs and PANI, BNCFs/PANI exhibited a substantially delocalized electronic state near the Fermi level, indicating enhanced charge transfer and transport. These results demonstrate that the synergistic interaction between BNCFs and PANI increases both the adsorption and catalysis of LiPSs, contributing to superior electrochemical performance.

## 3 Conclusion

In conclusion, we constructed a self‐supporting Li–S battery cathode based on bamboo‐like nitrogen‐doped carbon fibers coated with PANI as the sulfur host. The ultralong BNCFs were produced via a VLS growth process using melamine as a raw material, aluminum served as a catalyst yielding Al_4_C_3_ nanoparticles as intermediate products, which spontaneously self‐assembled into a continuous BNCFs film. Furthermore, synergistic interface engineering between BNCFs and PANI enabled the construction of a composite sulfur cathode that combines both physical confinement and chemical adsorption capabilities. The designed architecture features a three‐dimensional freestanding BNCFs framework that allows for rapid Li^+^/electron flow while physically constraining LiPSs. Simultaneously, the consistently coated PANI layer provides chemical binding of LiPSs via dynamic S–N interactions and enhances sulfur utilization through a reversible phenylenediamine/quinodiimide redox interconversion. By integrating nano‐confinement effects with polymer‐mediated interfacial chemistry, this dual‐confinement technique achieves excellent electrochemical performance: the BNCFs/S/PANI cathode exhibits an ultra‐low capacity degradation rate of 0.047% per cycle over 500 cycles at 1.0 A g^−1^, along with exceptional rate capability. This innovative design significantly improves the cycling stability and practical feasibility of Li–S batteries through the concurrent activation of redox kinetics.

## Author Contributions

**Fan Wang**: data curation, validation, writing – review and editing. **Terence Xiaoteng Liu**: funding acquisition, project administration, writing – review and editing. **Jie Yang**: conceptualization, methodology, validation, investigation, writing – original draft, data curation. **Enci Wang**: investigation, methodology. **Haile Qian**: investigation, validation. **Jiarui Huang**: resources, supervision, writing – review and editing.

## Funding

This work was supported by the UK‐Engineering Physics and Science Research Council (Grant No. EP/S032886/1) and the Scientific Research Project of Anhui Higher Education Institution (2024AH051843, 2024AH051839). The numerical calculations in this paper have been done on Hefei advanced computing center.

## Conflicts of Interest

The authors declare no conflicts of interest.

## Supporting information




**Supporting File**: advs76380‐sup‐0001‐SuppMat.docx.

## Data Availability

The data that support the findings of this study are available from the corresponding author upon reasonable request.
