# Supplementary material for: Freestanding Bamboo‐Like Nitrogen‐Doped Carbon Nanofibers/PANI Dual‐Conductive Cathodes via Interfacial Engineering for High‐Performance Lithium–Sulfur Batteries
Source: Adv Sci (Weinh). 2026 Jun 27;13(53):e76380. doi: 10.1002/advs.76380 (PMC13336891; doi:10.1002/advs.76380)
Supplement: Supplementary file 1 [file ADVS-13-e76380-s001.docx]

**Supporting information**

Freestanding Bamboo-Like Nitrogen-Doped Carbon Nanofibers/PANI Dual-Conductive Cathodes via Interfacial Engineering for High-Performance Lithium-Sulfur Batteries

*Jie Yang ^1^, Fan Wang ^2,^ *, Enci Wang ^1^, Haile Qian ^1^, Terence Xiaoteng Liu ^3,^ *, Jiarui Huang ^1,^ **

^1^ Key Laboratory of Functional Molecular Solids of the Ministry of Education, College of Chemistry and Materials Science, Anhui Normal University, Wuhu, Anhui 241002, P. R. China

^2^ School of Materials Science and Engineering, Tongling University, Tongling, Anhui 240000, P. R. China

^3^ School of Engineering, Physics and Mathematics, Faculty of Science and Environment

Northumbria University, Newcastle upon Tyne, Newcastle upon Tyne NE1 8ST, U.K.

**E-mail:** wangfan@tlu.edu.cn (Fan Wang), terence.liu@northumbria.ac.uk (T. X. Liu) and jrhuang@mail.ahnu.edu.cn (J. R. Huang)

**1 Experimental Section**

**Chemicals and materials**

Melamine (≥99%), Aluminum (Al), Polyaniline (PANI, ≥99.5%), ammonium persulfate ((NH_4_)_2_S_2_O_8_, ≥98%), and sulfur powder were sourced from Aladdin. Sulfuric acid (H_2_SO_4_, ≥95%) was supplied by Sinopharm Chemical Reagent Co., Ltd. Deionized water was used throughout the experiments, and all reagents were used as received without further purification.

**Synthesis of BNCFs–Al_4_C_3_**

BNCFs–Al_4_C_3_ was directly synthesized in one step by high–temperature catalysis. A multilevel structured BNCFs–Al_4_C_3_ carbon film composed of well–interwoven hollow carbon nanofibers was prepared by reacting melamine as the carbon source and metallic aluminum as a catalyst source in a high–temperature tube furnace at 1200 °C in a nitrogen atmosphere.

**Synthesis of BNCFs**

BNCFs–Al_4_C_3_ was placed in a solution of aqua regia and water (1:3), heated and reacted at 120 °C for 12 h, cooled, washed and dried.

**Synthesis of BNCFs/S**

The as–prepared BNCFs was cut into the desired discs pieces and weighed to calculate the material load. The disks were immersed in a mixed solution of S and carbon disulfide (S/CS_2_). After drying at 55 °C for 12 h to remove the CS_2_, sealed in a polytetrafluoroethylene vial, and maintained at 155 °C for 15 h. After naturally cooling to room temperature, the BNCFs/S cathode was obtained.

**Synthesis of BNCFs/S/PANI cathode**

Briefly, 0.1 g of BNCFs/S was dispersed in 60 mL of 0.43 M H_2_SO_4_ solution, followed by the addition of 15 μL aniline (C_6_H_7_N). Meanwhile, 0.568 g ammonium persulfate was dissolved in 40 mL of 0.43 M H_2_SO_4_ solution under stirring. The two solutions were then mixed together. After stirring in an ice bath for 12 h, the product was filtered and washed three times with deionized water and ethanol, and then dried at 60 °C to obtain the BNCFs/S/PANI composite.

**Assembly and electrochemistry testing of the symmetric cell**

First, Li_2_S_6_ solutions were prepared by dissolving stoichiometric amounts of Li_2_S and sulfur (1:5) in a mixed solvent of 1,3–dioxolane/1,2–dimethoxyethane (DME/DOL, 1:1 by volume) containing 1 M lithium bis(trifluoromethanesulfonyl)imide (LiTFSI) and 1 wt.% LiNO_3_. Both working and counter electrodes were BNCFs and BNCFs/PANI. The active material loading was controlled at approximately 1.6 mg cm^–2^. The identical working and counter electrodes of BNCFs and BNCFs/PANI were assembled into the symmetric cells with 40 μL Li_2_S_6_ electrolyte for the catalytic effect analysis. CV measurements of these symmetric cells were conducted between −0.8 and 0.8 V at a scan rate of 10 mV s^−1^.

**The Tafel tests**

The Tafel plots were conducted for the cells with the electrodes (BNCFs/PANI and BNCFs) as working electrodes, Li foils as counter electrodes, 20 μL Li_2_S_6_ electrolyte, scanning rate of 2 mV s^−1^, and the voltage range from 1.6−2.8 V.

**Measurement for the Li_2_S nucleation**

A Li_2_S_8_ solution (0.3 M) was obtained by mixing Li_2_S and S (1:7 molar ratio) in a DOL/DME solution (1:1 volume ratio) with vigorous stirring for 24 h. The prepared BNCFs/PANI and BNCFs membranes were directly used as electrodes. Celgard 2500 was chosen as the separator to separate the cathode and lithium anode. The prepared 15 μL Li_2_S_8_ solution and 15 μL Li–S battery electrolyte was added to the cathode side, followed by sequential placement of the separator and lithium foil. The assembled cells were galvanostatically discharged at 0.112 mA to a voltage of 2.10 V on the battery test system and then held constant at a voltage of 2.05 V in the electrochemical workstation until the test current was less than 10^–5^ A.

**Fabrication of Li–S pouch cell**

The BNCFs/S/PANI cathode and lithium anode were cut into 2.0×2.0 cm pieces. The sulfur loading of the cathode in the pouch cell was 1.3 mg cm^–2^. The E/S ratio was about 10 μL mg^–1^, and the thickness of the lithium belt anode was 100 μm. The separator and electrolyte were sandwiched between the tailored BNCFs/S/PANI and the lithium belt.

**2 Characterization methods**

The microstructure, dimensions, and morphology of the samples were analyzed using field emission scanning electron microscopy (FESEM, Hitachi Regulus S–8100, operating voltage 5–20 kV) equipped with an EDS (Hitachi Regulus S–8100, Oxford Instruments, 15 kV), as well as TEM (Hitachi HT–7700, acceleration voltage 120 kV). HRTEM images were acquired at an acceleration voltage of 200 kV. The phase structure and elemental valence states of the samples were determined by XRD (Rigaku SmartLab D8 Advance, wavelength λ=1.54060 Å) and XPS (Thermo Scientific K–Alpha), respectively. Specific surface area and pore size distribution were measured using a Micromeritics ASAP 2460 analyzer (Shanghai Micromeritics Instrument Co., Ltd., with N_2_ as the adsorbate). Thermogravimetric analysis was performed on a Setaram Labsys Evo SDT Q600 thermal analyzer under flowing nitrogen or air atmosphere, with a heating rate of 10 °C min^–1^ from room temperature to 700 °C, to determine the sulfur content in the composites. Raman spectra were recorded using a Renishaw in–via system with a laser wavelength of 532 nm, a measured laser power of 5 mW at the sample position, and a spectral acquisition range of 2000–100 cm^–1^.

**3 Electrochemical measurements**

The electrochemical properties of BNCFs/S and BNCFs/S/PANI composites as self–supported cathode materials for Li–S batteries were investigated (CR2032). The batteries were assembled in an argon filled glove box (O_2_ < 0.01 ppm, H_2_O < 0.01 ppm, Michelona, Super 1220/750/900). The counting/reference electrode was made of lithium foil (Aladdin Reagent Shanghai Co., Ltd.). The electrolyte was 1 M lithium bis(trifluoromethane)sulfonamide salt (LiTFSI, Sigma–Aldrich) with DOL and DME as mixed solvents in the ratio of 1:1 by volume, containing 1% LiNO_3_ additive. The sulfur loading of BNCFs/S and BNCFs/S/PANI electrodes was controlled to be about 1.2 mg cm^−2^, and the electrolyte–to–sulfur ratio was controlled to be ca. 16.0 μL mg^−1^. A higher areal sulfur loading of the BNCFs/S/PANI cathode fabricated using the sample was controlled at approximately 4.1 mg cm^–2^ and the electrolyte–to–sulfur ratio was also controlled at ca. 5.0 uL mg^−1^. The discharge–charge tests were performed on a Neware battery test system at a constant temperature of 25 °C, with a potential range of 1.6 to 2.8 V. CV curves and electrochemical impedance spectra (EIS, 0.01 Hz to 100 kHz) were performed on an electrochemical workstation.

**4 Density functional theory calculations**

The first–principles simulations of PNAI, graphene and graphene–PNAI were carried out using Perdew−Burke−Ernzerhof generalized gradient approximation (GGA) based the Vienna ab Initio simulation package (VASP).^[1, 2]^ The projector augmented–wave (PAW) pseudopotential were applied to spin–unrestricted geometry optimizations. ^[3, 4]^ The cutoff energy for the plane wave basis was set to 400 eV. The convergence threshold of the electronic self–consistency was specified as 1.0×10^–4^ eV, and the total energy change of the whole catalyst system between two ionic relaxation steps was designated as less than 0.05 eV. The 2D models were built using VESTA program.^[5]^ The adsorption energy (*E_ads_*) was expressed as

$$E_{ads}=E_{(surf+mol)}-E_{(surf)}-E_{(mol)}$$

where $E_{(surf)}$ and $E_{(mol)}$ are the single point energies of substrates and isolated molecules, respectively, and $E_{(surf+mol)}$ represents the energy of the combined systems upon adsorption

**Supporting Figures**

**Figure S1** The digital images of pure BNCFs.

**Figure S2** SEM images of (a) BNCFs–Al_4_C_3_, (b) BNCFs, (c) BNCFs/S, and (d) BNCFs/S/PANI. TEM images of (e) BNCFs–Al_4_C_3_, (f) BNCFs, (g) BNCFs/S, and (h) BNCFs/S/PANI. (i), and (j) TEM image and corresponding elemental mapping images of BNCFs.

**Figure S3** TEM images of BNCFs/S/PANI with different aniline dosages: (a) 5 μL; (b) 30 μL.

**Figure S4** EDS spectrum of (a) BNCFs–Al_4_C_3_, (b) BNCFs, and (c) BNCFs/S/PANI.

**Figure S5** The digital images of pure BNCFs, BNCFs/S, and BNCFs/S/PANI electrodes.

**Figure S6** (a) Raman spectrum of BNCFs/S and BNCFs/S/PANI. The deconvoluted Raman spectra from the Raman data in panel (b) BNCFs/S/PANI and (c) BNCFs/S at the D and G band. (d) N_2_ adsorption/desorption isotherm of BNCFs/S and BNCFs/S/PANI. The insets are the corresponding [pore size distribution](https://www.sciencedirect.com/topics/chemistry/pore-size-distribution)s.

**Figure S7** High–resolution XPS spectra of BNCFs–Al_4_C_3_: (a) survey spectra, (b) C 1s, and (c) N 1s. High–resolution XPS spectra of BNCFs: (d) survey spectra, (e) C 1s, and (f) N 1s.

**Figure S8** High–resolution XPS spectra of BNCFs/S/PANI: (a) survey spectra, and (b) O 1s.

**Figure S9** (a) Initial five cycles CV curves of BNCFs/S at a scan rate of 0.1 mV s^−1^. (b) Rate performance of BNCFs/S/PANI-5 and BNCFs/S/PANI-30. (c) Galvanostatic charge/discharge profiles of BNCFs/S. Cycling performance of BNCFs/S and BNCFs/S/PANI at (d) 2.0 A g^−1^ and (e) 5.0 A g^−1^.

**Figure S10** (a) Cycling performance of BNCFs/S with high S loadings at 0.5 A g^−1^. (b) Cycling performance of the pouch cell using BNCFs/S at 0.1 A g^−1^.

**Figure S11** Illustration of pouch cell assembly.

**Figure S12** Tafel plots derived from Peaks 2.

**Figure S13** Nyquist plot of BNCFs/S/PANI and BNCFs/S after 500 cycles. The insets are the corresponding simulation circuit diagram.

**Figure S14** Nyquist plots of BNCFs/S/PANI before and after folding. The insets are the corresponding simulation circuit diagram.

**Figure S15** XPS spectra of fresh BNCFs/PANI–Li_2_S_6_ and BNCFs/PANI.

**Figure S16** SEM images of (a) BNCFs/S, and (b) BNCFs/S/PANI after 500 cycling test.

**Figure S17** XRD pattern of BNCFs/S/PANI cathode after cycling.

**Figure S18** Photographs of the separator of disassembled coin batteries: BNCFs/S and BNCFs/S/PANI.

**Figure S19** SEM images of the cycled Li metal anodes of batteries with (a) BNCFs/S, and (b) BNCFs/S/PANI cathodes (insets: digital photographs of Li metal anodes).

**Table S1** The atomic percentage of each element obtained from XPS anlysis of BNCFs/PANI.

| Sample | C (%) | O (%) | N (%) |
| --- | --- | --- | --- |
| BNCFs/PANI | 86.11 | 9.81 | 4.08 |

**Table S2** Comparison of the capacity retention of previous reports with this study.

| Materials | Current density (A g^−1^) | Cycle number  (time) | | Capacity  (mAh g^−1^) | Refs. |
| --- | --- | --- | --- | --- | --- |
| WLC–CNTs | 0.167  0.335 | 100  300 | 692.0  547.0 | | [36] |
| iPANI@rGO–CNTs | 0.837  3.350 | 300  500 | 815.6  620.3 | | [39] |
| High O_x_–SWCNTs | 0.167 | 100 | 805.0 | | [58] |
| S@CCGM | 0.335  0.837 | 300  300 | 559.3  482.4 | | [59] |
| P-Mn_3_O_4−_*_x_* | 1.675 | 250 | 693.7 | | [60] |
| SC@TiC/rGO–S | 0.167 | 300 | 852.1 | | [61] |
| CP/g-C_3_N_4_@CC@S | 1.675 | 500 | 516.9 | | [62] |
| i–CMO/S | 1.675 | 400 | 611.3 | | [63] |
| S/CF | 0.335  1.675 | 100  100 | 737.0  530.0 | | [64] |
| BNCFs/S/PANI | 0.2  1.0 | 100  500 | 852.4  527.1 | | This work |

The Li^+^ diffusion process can be depicted by EIS result as follow:

$D_{ion}=\frac{R^{2}T^{2}}{2A^{2}n^{4}F^{4}C^{2}\sigma^{2}}$

$Z^{'}=R+ \sigma\omega^{-1/2}$

**Table S3** *D*_Li_^+^ calculation results of three electrodes (Unit: cm^2^ s^−1^)

|  | *D*_Li_^+^ |
| --- | --- |
| BNCFs/S/PANI | 6.28×10^-15^ |
| BNCFs/S | 2.59×10^-15^ |

**Table S4** Simulation model with the estimated values.

| *R*(Ohm) | *R*_1_ | *R*_2_ | *R*_ct_ |
| --- | --- | --- | --- |
| BNCFs/S/PANI (before cycling test) | 2.7 | − | 56.2 |
| BNCFs/S/PANI (after500 cycles) | 6.1 | 48.6 | 26.7 |
| BNCFs/S (before cycling test) | 2.7 | − | 65.7 |
| BNCFs/S (after 500 cycles) | 5.3 | 46.5 | 71.0 |

**Table S5** Simulation model with the estimated values.

| *R*(Ohm) | *R*_1_ | *R*_ct_ |
| --- | --- | --- |
| BNCFs/S/PANI (before folding) | 0.88 | 40.5 |
| BNCFs/S/PANI (after folding) | 0.95 | 31.2 |

**References**

[1] J. P. Perdew, K. Burke, M. Ernzerhof, Generalized gradient approximation made simple, *Phys. Rev. Lett.* **1996,** *77,* 3865−3868.

[2] G. Kresse, J. Furthmuller, Efficient iterative schemes for ab initio total−energy calculations using a plane−wave basis set, *Phys. Rev. B* **1996,** *54* , 11169−11186.

[3] G. Kresse, D. Joubert, From ultrasoft pseudopotentials to the projector augmented−wave method, *Phys. Rev. B 59,* **1999,** 1758−1775.

[4] P.E. Blöchl, Projector augmented−wave method, *Phys. Rev. B* **1994,** *50,* 17953−17979.

[5] K. Momma, F. Izumi, VESTA 3 for three−dimensional visualization of crystal, volumetric and morphology data, *J. Appl. Crystallogr.* **2011,** *44,* 1272-1276.
